# Minimal kyphotic progression following pediatric vertebral fractures: implications for reduced radiographic surveillance

**DOI:** 10.1007/s00402-026-06482-2

**Published:** 2026-08-26

**Authors:** Sebastian Wegmann, Maximilian Lenz, Tamara Babasiz, Peer Eysel, Lars Peter Mueller, Maximilian Weber

**Affiliations:** Department of Orthopedics, Trauma Surgery, Plastic and Aesthetic Surgery, University Hospital Cologne, Cologne, Germany

**Keywords:** Spine trauma, Radiograph, Fracture detection, Children large

## Abstract

**Introduction:**

Pediatric spinal fractures are rare injuries (14.24 per 100,000 per year), representing only 0.6–3% of all spinal injuries. While most stable compression fractures are managed conservatively, concerns exist regarding progressive vertebral collapse and kyphotic deformity. Unlike extensive adult literature, pediatric-specific data on radiographic progression following conservative management remain limited, creating uncertainty about the clinical utility of routine follow-up imaging in this radiosensitive population.

**Materials and methods:**

Retrospective single-centre cohort study of 54 pediatric patients (mean age 12.3 years) with traumatic vertebral body fractures diagnosed between 2012 and 2024. Kyphosis angles were measured using Cobb methodology at baseline and follow-up intervals (1, 3, and 6 weeks). The primary outcome was the change in kyphotic angle; secondary outcomes included the identification of predictors of progression. Paired t-tests and multivariable linear regression analysis were performed.

**Results:**

Kyphosis angles remained essentially unchanged across all follow-up intervals (ΔFU1 + 0.19°, ΔFU2 + 1.27°, ΔFU3 − 0.14°), with no statistically significant differences detected (*p* > 0.05 at all timepoints). No clinical or radiographic factors predicted progression of kyphosis. Only three patients (5.6%) required treatment escalation. Subgroup analyses revealed no significant differences across fracture types, treatment modality, or injury characteristics.

**Conclusion:**

Pediatric patients with stable compression fractures demonstrate minimal kyphotic progression regardless of treatment approach. Routine radiographic surveillance provides little clinical value and exposes children to unnecessary ionising radiation. The present study suggests that routine radiographic follow-up may not be necessary in carefully selected patients with stable vertebral compression fractures who remain clinically asymptomatic. Nevertheless, these findings should be interpreted in light of the retrospective study design and the limited sample size. Confirmation in larger prospective studies is needed before changes to routine follow-up protocols can be recommended.

## Introduction

Spinal fractures in children remain uncommon injuries, with a global incidence of approximately 14.24 per 100,000 per year [1]. Traumatic structural vertebral injuries represent only 0.6% to 3% of all pediatric and adolescent spinal injuries, making them rare clinical encounters [2]. Nevertheless, their management requires meticulous clinical and radiological evaluation due to the fundamentally different biomechanical and anatomical characteristics of the developing spine compared to those of the adult spine. The pediatric vertebral column exhibits increased elasticity, a greater disc-to-vertebral body ratio, and elevated cartilaginous content throughout the spinal unit. These features confer both protective advantages—increased resilience to high-energy trauma—and inherent vulnerabilities to subtle ligamentous instability and progressive angular deformity [3]. Consequently, injury patterns follow a precise age-dependent distribution: compression fractures of the thoracic and lumbar spine predominate in children over 10 years of age, whereas cervical spine injuries occur more frequently in younger children, mainly attributable to the disproportionate head-to-body ratio and incomplete neuromuscular development [4]. 

The thoracolumbar junction represents the most common site of injury in pediatric vertebral fractures, with simple compression-type injuries (AO Spine Type A) constituting the predominant fracture pattern [5]. In the absence of neurological deficits or disruption of the posterior ligamentous complex (PLC), most of these fractures are managed conservatively. Conservative management typically involves avoiding end-range rotational and inclination movements and maximizing spine unloading. This can be facilitated by using a coreset orthosis. However, even when initially appearing stable, a subset of patients may develop progressive vertebral body height loss and an increase in local kyphosis—referred to as post-traumatic kyphotic deformity or secondary collapse. This reflects a delayed biomechanical failure of the vertebral body under physiological loading, particularly in fractures involving the anterior column of the spine. While adult literature has comprehensively investigated post-traumatic kyphosis, establishing clear associations between deformity progression and fracture morphology, patient age, and treatment modality, the pediatric population remains underrepresented in these analyses, especially in the context of conservative therapy [6]. For secondary post‑traumatic deformities, no classification system currently exists that delineates treatment strategies or provides standardised criteria for evaluating local and global spinal alignment [7]. Consequently, clinical centres have adopted follow-up imaging protocols from adult practice, typically performing serial radiographs at regular intervals (e.g., 1, 3, and 6 weeks post-injury) to detect progressive deformity and inform treatment escalation decisions [8–10]. At our institution, follow-up radiographs are routinely obtained after approximately 1, 3, and 6 weeks.

This gap in pediatric-specific evidence on the natural history and radiographic progression poses a dilemma for clinicians: the potential clinical benefit of interval imaging must be weighed against the cumulative radiation exposure from repeated radiological surveillance in a uniquely radiosensitive population.

The primary objective was to quantify changes in the kyphotic angle over the follow-up time, assess the incidence and extent of post-traumatic vertebral collapse, and identify patient or injury characteristics that reliably predict progression. We hypothesised that radiological follow-up examinations in cases of stable compression fractures, classified according to the AO-Spine system, are unnecessary from a radiation protection standpoint and therefore not required in children and adolescents.

## Materials and methods

### Study design

This study is a retrospective, single-cent cohort analysis conducted at a Level 1 Trauma Centre and a Pediatric Trauma Reference Centre within the local Trauma network. The investigation is based on a review of electronic medical records (Orbis system) and radiological imaging of pediatric and adolescent patients aged 0 to 18 years diagnosed with traumatic vertebral body fractures.

Eligible for inclusion were patients with at least one trauma-related vertebral body fracture diagnosed between January 1, 2012, and December 31, 2024. Inclusion criteria required complete clinical documentation and radiographic records, including at least one follow-up imaging study. Exclusion criteria comprised pathological fractures (e.g., due to tumor, infection, or osteoporosis), congenital spinal anomalies, and incomplete clinical or radiological documentation. The conservative treatment consisted of avoiding end-range rotational and spinal inclination movements, possible spine unloading, and physiotherapy; a corset therapy was not used in the presented cases.

### Study population

An initial keyword search of the electronic health records and an ICD-10 codes search identified 140 pediatric patients with vertebral body fractures who received treatment at the study centre within the defined period. After applying the exclusion criteria, 86 cases were removed due to incomplete data, pathologic fracture aetiology, or absence of follow-up imaging. A total of 54 cases met all inclusion criteria and were included in the final analysis. The study cohort reflects the spectrum of pediatric vertebral fractures treated at our institution during the study period. Patients with different fracture morphologies and treatment strategies were therefore included to describe routine clinical practice. However, the primary clinical question of this study focuses on the radiographic course of stable vertebral compression fractures managed conservatively.

### Data collection

Clinical parameters recorded at baseline included the presence of back pain, neurological deficit, and radiographic spinal canal compromise. The treatment modality was classified as either conservative or operative. Fractures were categorised using the AO Spine Classification system based on radiographic and cross-sectional imaging.

The primary radiographic variable was the kyphosis angle, measured on lateral spine radiographs at the time of injury and at each available follow-up. Follow-up radiographs were categorised chronologically as Follow-Up 1 (FU1), Follow-Up 2 (FU2), and Follow-Up 3 (FU3). The change in kyphosis at each follow-up was calculated as the difference between the follow-up angle and the initial angle at presentation. The interval between injury and follow-up was recorded in days based on imaging timestamps.

The primary outcome was the change in kyphosis at the first follow-up (ΔFU1). Secondary outcomes included changes in kyphosis at later follow-up examinations (ΔFU2 and ΔFU3) and the identification of potential predictors of kyphosis progression.

### Radiological measurement methods

Kyphotic angle measurements were conducted using digital imaging software (IMPAX, Agfa HealthCare) based on available CT, MRI, or plain radiographs. The initial kyphosis angle was measured at the time of trauma, while subsequent measurements were primarily based on lateral radiographs during follow-up.

The Cobb angle measurement was used as the standard technique to determine sagittal spinal curvature, as it presents sufficient inter-rater and intra-rater reliability [11, 12]. Measurements were performed between lines perpendicular to the upper endplate of the superior vertebra and the lower endplate of the inferior vertebra at the fracture site. All Cobb angle measurements were performed by trained investigators using a standardized measurement protocol. Formal intra- and interobserver reliability analyses were not performed.

### Statistical analysis

Descriptive statistics were used to summarise demographic, clinical, and radiographic variables. Continuous data are reported as means with standard deviations, and categorical data as frequencies and percentages. Changes in kyphosis between the initial examination and each follow-up (FU1–FU3) were analysed using paired t-tests. Differences in kyphosis progression (ΔFU) between clinical or radiographic subgroups (e.g., treatment modality, AO fracture type, spinal canal compromise, neurological deficit, pain, sex) were assessed using t-tests or one-way ANOVA as appropriate. To explore potential predictors of kyphosis change, a multivariable linear regression model was performed with ΔFU as the dependent variable and clinically relevant baseline factors (initial kyphosis angle, age, follow-up interval, spinal canal compromise, neurological deficit, and pain) as independent variables. Statistical significance was defined as *p* < 0.05.

## Results

Of 140 patients identified with suspected vertebral body fractures, 54 pediatric patients (25 female, 29 male) met all inclusion criteria and were included in the final analysis. The cohort had a mean age of 12.3 years (± 4.2). The majority of injuries were classified as AO Spine Type A1 (40/54; 74.1%), followed by A4 (3/54; 5.6%), B2 (3/54; 5.6%), A2 (2/54; 3.7%), A3 (2/54; 3.7%), C-type fractures (2/54; 3.7%), and A0 fractures (2/54; 3.7%). Fracture regions were predominantly thoracic (32/54; 59.3%), followed by lumbar injuries (19/54; 35.2%), while cervical fractures were rare (3/54; 5.6%) (Table 1). Initial management was conservative in 39 patients (72.2%) and operative in 15 patients (27.8%). Spinal canal compromise was present in 5 children (9.3%), neurological deficits in 4 (7.5%), and 32 patients (59.3%) reported back pain at presentation.

All patients underwent at least one radiographic follow-up (FU). Nineteen patients had one follow-up examination, eighteen had two, thirteen had three, and four had four or more follow-ups. The mean (± SD) time from injury to imaging was 13.9 ± 16.9 days for the first follow-up (FU1, *n* = 52), 46.5 ± 42.7 days for the second (FU2, *n* = 34), and 133.6 ± 167.1 days for the third (FU3, *n* = 17). Therapy adjustments during follow-up were rare. After FU1, two patients required escalation of treatment: one underwent ventral stabilisation, and the other received lumbopelvic fixation (instrumentation of L4–L5 with extension to the sacrum). No therapy changes occurred FU2. After FU3, one patient required operative revision, which consisted of extending a prior L2–L4 fusion to L2–L5.

The initial kyphosis angle measured 14.71° ± 8.73° (*n* = 54). At the first follow-up (FU1), the kyphosis angle was 14.70° ± 8.68° (*n* = 52), demonstrating an almost identical distribution to the baseline measurement. At later follow-ups, the kyphosis angles were 14.80° ± 8.40° at FU2 (*n* = 34) and 16.10° ± 12.40° at FU3 (*n* = 17). Changes in kyphosis were minimal across all timepoints. The ΔFU1 averaged + 0.19° ± 5.60°, ΔFU2 averaged + 1.27° ± 7.80°, and ΔFU3 averaged − 0.14° ± 7.50°.

Paired t-tests showed no statistically significant differences between initial and follow-up measurements (initial vs. FU1: t = − 0.24, *p* = 0.8097; initial vs. FU2: t = − 0.88, *p* = 0.3866; initial vs. FU3: t = 0.07, *p* = 0.9483). To assess whether kyphosis changed over follow-up, additional paired analyses were performed among patients with consecutive radiographs. No statistically significant differences were found between FU1 and FU2 (*p* = 0.2410), between FU1 and FU3 (*p* = 0.4808), or between FU2 and FU3 (*p* = 0.9389). Thus, the kyphosis angle remained stable not only relative to the baseline but also across all sequential follow-up time points.

Subgroup analyses likewise revealed no statistically significant differences in kyphosis progression. ΔFU1 did not differ across AO fracture types (*p* = 0.0579), between conservative and operative treatment (*p* = 0.3229), between males and females (*p* = 0.5669), or between patients with versus without spinal canal compromise (*p* = 0.6604). No significant differences were observed for neurological deficit (*p* = 0.1580) or pain at presentation (*p* = 0.4735).

The multivariable linear regression model included initial kyphosis angle, follow-up interval, age, spinal canal compromise, neurological deficit, and pain. None of the variables reached statistical significance. However, the initial kyphosis angle showed the most significant regression coefficient (β = − 0.217, *p* = 0.0687), indicating a nonsignificant tendency toward slightly greater kyphosis reduction in patients with higher initial angles. The neurological deficit showed the second-largest coefficient (β = 5.79, *p* = 0.1350), indicating that children with a neurological deficit were estimated to have a 5.79° greater change in kyphosis compared with those without a deficit. However, this effect did not reach statistical significance. The negative coefficient of the initial kyphosis angle (β = − 0.217) indicates that children who presented with a more pronounced initial deformity tended to show slightly greater improvement over the follow-up period. In contrast, the positive coefficient associated with neurological deficit (β = 5.79) suggests an estimated tendency toward greater kyphosis progression among children with neurological impairment. All other predictors showed small coefficients with large p-values (follow-up interval, *p* = 0.9483; age, *p* = 0.4630; spinal canal compromise, *p* = 0.6901; pain, *p* = 0.3571).

**Table 1:** Distribution of Exact Fracture Levels

Region	Vertebral Level	Count (*n*)	% of Total	Regional Subtotal
Cervical Spine	C5	2	3.7%	**3 (5.6%)**
	C7	1	1.9%	
Thoracic Spine	T1	4	7.4%	**31 (57.4%)**
	T2	1	1.9%	
	T3	1	1.9%	
	T4	2	3.7%	
	T5	2	3.7%	
	T6	5	9.3%	
	T7	3	5.6%	
	T8	3	5.6%	
	T9	3	5.6%	
	T11	3	5.6%	
	T12	4	7.4%	
Lumbar Spine	L1	7	13.0%	**19 (35.2%)**
	L2	6	11.1%	
	L3	3	5.6%	
	L4	2	3.7%	
	L5	1	1.9%	
Total		54	100%	

Exact distribution of vertebral fracture levels across the cervical, thoracic, and lumbar spine. Values indicate exact values and the percentage of the whole.

## Discussion

The most important finding of this study is that pediatric patients with stable vertebral compression fractures demonstrate minimal progression of kyphotic deformity during follow-up, regardless of treatment modality or injury characteristics. Across all follow-up intervals, the kyphotic angle remained essentially unchanged from baseline (ΔFU1 + 0.19°, ΔFU2 + 1.27°, ΔFU3 − 0.14°), with no statistically significant differences detected at any time point. Furthermore, no baseline clinical or radiographic factors—including AO fracture type, treatment approach, spinal canal compromise, neurological deficit, or pain—were identified as significant predictors of kyphosis progression. These findings suggest that routine radiological follow-up in conservatively managed stable compression fractures may be unnecessary from both a clinical and radiation protection standpoint, supporting a more selective, symptom-driven imaging strategy in this vulnerable pediatric population.

### Radiographic stability and clinical implications

The remarkable stability of sagittal alignment observed in this cohort challenges the conventional practice of scheduled serial radiographic surveillance following pediatric spinal trauma. Despite theoretical concerns about progressive vertebral body height loss and secondary collapse in the developing spine, the data demonstrate that such progression is rare in appropriately selected patients with stable injury patterns. Only three patients (5.6%) required treatment escalation across all follow-up periods, and these revisions occurred in cases where clinical symptoms or initial injury severity would have warranted closer monitoring regardless of routine protocols.

This observation has important implications for clinical practice. Pediatric patients are particularly vulnerable to the cumulative effects of ionizing radiation, with an increased lifetime cancer risk compared to adults exposed to equivalent doses [13, 14]. Current practice commonly includes routine follow-up radiographs at predefined intervals, such as 1, 3, and 6 weeks after injury. However, our findings suggest that, in carefully selected patients with stable vertebral compression fractures, a more selective, symptom-driven imaging strategy may reduce unnecessary radiation exposure while remaining consistent with the ALARA (As Low As Reasonably Achievable) principle. Such an approach may also reduce unnecessary healthcare utilization without compromising patient safety [15]. 

### Incidence and fracture patterns

Consistent with existing literature, this study confirms that pediatric vertebral fractures are uncommon injuries, with the thoracolumbar region being the most frequently affected site [16, 17]. The predominance of AO Spine Type A1 compression fractures (74.1%) reflects the characteristic injury mechanism and biomechanical properties of the growing spine, which demonstrates greater elasticity and cartilaginous content compared to adult vertebrae. The age-dependent distribution of fracture patterns was evident, with thoracic and lumbar injuries more common in older children—consistent with the transition toward adult-like biomechanical characteristics as skeletal maturity approaches [18]. 

### Predictors of kyphosis progression

The multivariable regression analysis revealed no statistically significant predictors of kyphotic change, though two variables showed nonsignificant trends worth discussing. The initial kyphosis angle showed the strongest coefficient (β = − 0.217, *p* = 0.0687), suggesting a mild tendency toward spontaneous correction in patients with greater baseline deformity. This finding is intriguing and may reflect the spine’s inherent capacity for remodelling during growth, particularly in younger patients with greater remaining skeletal development. The Hueter-Volkmann principle—which describes asymmetric growth in response to mechanical loading—may contribute to the gradual correction of mild-to-moderate kyphotic deformities over time in the pediatric population [19]. 

The absence of statistically significant predictors should be interpreted with caution, as the limited sample size and the small number of patients with uncommon fracture patterns or neurological deficits reduce the statistical power to detect potentially relevant associations.

Neurological deficit at presentation showed the second-largest coefficient (β = 5.79, *p* = 0.1350), indicating a trend toward greater kyphosis progression in these patients. However, the small number of affected individuals (*n* = 4, 7.5%) limits the statistical power to detect this effect. This observation warrants clinical attention, as neurological compromise typically indicates more severe injury patterns with potential posterior ligamentous complex disruption or spinal canal compromise—factors that may predispose to late instability. Patients presenting with neurological deficits clearly require closer surveillance regardless of initial radiographic appearance, and this subgroup may not be appropriate candidates for reduced imaging protocols.

### Treatment approach and escalation

The majority of patients (72.2%) were managed conservatively, reflecting appropriate clinical decision-making based on injury stability and neurological status. The conservative treatment in our clinic, presented in the materials and methods section, consisted of a regimen similar to that recommended for adults; however, patients did not receive a corset orthosis. Literature for children in the case of traumatic fractures is not present; for adults, the results show no statistically significant difference after therapy with or without orthosis [20]. Orthotic bracing may offer potential benefits in pediatric patients and should thus be investigated in future studies. The low rate of treatment escalation (5.6% overall), which further supports the generally benign natural history of stable compression fractures in children. The three patients requiring surgical revision all had features that, in retrospect, might have predicted higher risk: two underwent stabilisation procedures following the first follow-up, suggesting that initial injury severity or clinical presentation warranted consideration of early intervention. The single patient who required fusion extension after the third follow-up had undergone prior operative management, indicating a complex injury pattern from the outset.

These observations underscore the importance of careful initial assessment and patient selection rather than routine surveillance. Clinical parameters—including mechanism of injury, initial neurological status, posterior element involvement, and initial deformity—should guide individualised follow-up strategies rather than blanket protocols applied uniformly to all compression fractures.

### Comparison with adult literature and classification challenges

While the adult literature has extensively examined post-traumatic kyphosis and established clear thresholds for intervention, pediatric-specific data remain limited [21–23]. The absence of a validated classification system for secondary post-traumatic deformities in children further complicates treatment decision-making and outcome assessment. The current study demonstrates that direct extrapolation from adult guidelines may not be appropriate, given the distinct biomechanical properties, remodelling potential, and injury patterns characteristic of the developing spine.

The AO Spine Classification system, while useful for initial injury characterization, was not designed to predict progressive deformity in children. Future research should focus on developing pediatric-specific prognostic tools that incorporate skeletal maturity, growth potential, and injury mechanism to identify better the small subset of patients at risk for clinically significant progression.

### Limitations

Several limitations should be considered when interpreting our findings. The retrospective single-centre design inevitably introduces the potential for selection bias and limits the generalizability of the results. Furthermore, only patients with available follow-up imaging were included in the final analysis, resulting in the exclusion of a substantial proportion of the initially identified cohort. Patients with an uncomplicated clinical course may have been discharged without further radiographic follow-up, whereas those with persistent symptoms or more complex injuries may have been monitored more closely. Consequently, the direction and magnitude of this potential selection bias cannot be determined retrospectively. Although this cohort represents a considerable number of pediatric vertebral fractures from a single institution, the overall sample size remains limited. As a result, the study is unlikely to detect very uncommon events, such as clinically relevant late kyphotic progression. Therefore, the absence of significant progression in our cohort should not be interpreted as evidence that delayed deformity cannot occur. Instead, our data suggest that such progression is uncommon in children with stable vertebral compression fractures treated according to our institutional protocol.

Additionally, although Cobb angle measurements are generally considered reliable, formal intra- and interobserver reliability analyses were not performed in the present study. Consequently, measurement variability cannot be excluded, particularly across different imaging modalities (radiographs, CT, MRI) [24]. Further, the study did not systematically assess functional outcomes, pain scores, return to activity, or quality of life measures at follow-up, which may be more clinically relevant than radiographic parameters alone. Long-term follow-up extending into adulthood would be valuable to determine whether minor radiographic changes observed in childhood translate into clinically significant degenerative changes or functional limitations later in life.

### Future directions and clinical recommendations

Our findings indicate that routine follow-up imaging may not be necessary in every child with a stable vertebral compression fracture. However, given the retrospective nature of this study and the limited cohort size, these observations should be interpreted with appropriate caution. Larger prospective multicentre studies are needed to determine whether a more selective imaging strategy can be adopted safely without overlooking the rare patient who develops clinically relevant deformity during follow-up.

Specific high-risk features that should prompt radiographic surveillance include: neurological deficit at presentation, significant spinal canal compromise, posterior ligamentous complex injury, multiple-level fractures, younger age with substantial remaining growth, and mechanism suggesting high-energy trauma [25]. For low-risk patients—typically older children and adolescents with isolated AO Type A1 fractures, no neurological deficit, and minimal initial kyphosis—clinical follow-up alone may be sufficient, with imaging reserved for new or worsening symptoms.

## Conclusion

This study demonstrates that pediatric patients with stable vertebral compression fractures, particularly those classified as AO Type A and managed conservatively, exhibit minimal radiographic progression of kyphosis over time. The absence of significant deformity progression across all follow-up intervals, combined with the extremely low rate of treatment escalation, challenges the routine use of scheduled radiographic surveillance in this population. From a radiation protection perspective, routine follow-up imaging appears to provide little clinical value in appropriately selected patients without new or progressive symptoms. These findings support consideration of a more selective imaging strategy toward individualised, symptom-driven radiological surveillance strategies that balance patient safety, clinical effectiveness, and judicious healthcare resource utilisation. Future prospective studies with larger cohorts and standardized protocols are needed to validate these findings and establish evidence-based guidelines for follow-up imaging in pediatric spinal trauma.

## Data Availability

The data are available upon reasonable request from the corresponding author.
